# The in vivo reduction of afferent facilitation induced by low frequency electrical stimulation of the motor cortex is antagonized by cathodal direct current stimulation of the cerebellum

**DOI:** 10.1186/s40673-016-0053-3

**Published:** 2016-08-30

**Authors:** Nordeyn Oulad Ben Taib, Mario Manto

**Affiliations:** 1 Department of Neurosurgery, CHU Saint-Pierre, Rue Haute, 1000 Bruxelles, Belgium; 2 Unité d’Etude du Mouvement (UEM), FNRS, Neurologie ULB-Erasme, 808 Route de Lennik, 1070 Bruxelles, Belgium; 3 Service des Neurosciences, Université de Mons, 7000 Mons, Belgium

**Keywords:** Neuromodulation, Plasticity, Motor cortex, Cerebellum, Direct current stimulation, In vivo

## Abstract

**Background:**

Low-frequency electrical stimulation to the motor cortex (LFSMC) depresses the excitability of motor circuits by long-term depression (LTD)-like effects. The interactions between LFSMC and cathodal direct current stimulation (cDCS) over the cerebellum are unknown.

**Methods:**

We assessed the corticomotor responses and the afferent facilitation of corticomotor responses during a conditioning paradigm in anaesthetized rats. We applied LFSMC at a frequency of 1 Hz and a combination of LFSMC with cDCS.

**Results:**

LFSMC significantly depressed both the corticomotor responses and the afferent facilitation of corticomotor responses. Simultaneous application of cDCS over the cerebellum antagonized the depression of corticomotor responses and cancelled the depression of the afferent facilitation.

**Conclusion:**

Our results demonstrate that cDCS of the cerebellum is a potent modulator the inhibition of the motor circuits induced by LFSMC applied in vivo. These results expand our understanding of the effects of cerebellar DCS on motor commands and open novel applications for a cerebellar remote control of LFSMC-induced neuroplasticity. We suggest that the cerebellum acts as a neuronal machine supervising not only long-term potentiation (LTP)-like effects, but also LTD-like effects in the motor cortex, two mechanisms which underlie cerebello-cerebral interactions and the cerebellar control of remote plasticity. Implications for clinical ataxiology are discussed.

## Background

Direct current stimulation (DCS) is growingly applied to understand the roles of the cerebellum on the sensorimotor or cognitive operations, and to modulate the effects of the cerebellum over the cerebral cortex in ataxic disorders [1]. Anodal DCS (aDCS) of the cerebellum reinforces the inhibition exerted by the Purkinje neurons over cerebellar nuclei, whereas cathodal DCS (cDCS) of the cerebellum induces a disinhibition of cerebellar nuclei, which physiologically excite thalamic targets amongst others. In other words, aDCS decreases the excitatory drive exerted by cerebellar nuclei, with opposite effects of cDCS [2]. The polarity-specific modulation of cerebellar-motor cortex connectivity is currently mainly explained by changes of the activity of the cerebello-thalamo-cortical pathways [2]. These tracts represent the best candidates for gating of the information flow from the cerebellum to the cerebral cortex [3]. Cerebellar nuclei project in particular to thalamic nuclei which target themselves the layers IV and V of the primary motor cortex, whose horizontal neuronal connections represent a substrate for map reorganization during plasticity [4].

It has been demonstrated that low frequency stimulation of the motor cortex (≤1 Hz) with repeated TMS (rTMS) exerts powerful inhibitory effects on corticospinal excitability by synaptic mechanisms similar to long-term depression (LTD) [5]. These effects are site-specific, unaffecting the contralateral motor cortex. The increased inhibition of the motor cortex might participate in the deficits observed in cerebellar disorders [6, 7]. Since cDCS of the cerebellum disinhibits cerebellar nuclei, this technique might be useful to antagonize the decreased excitability of the motor cortex in cerebellar patients. We tested the hypothesis that cDCS of the cerebellum counteracts the inhibitory effects exerted by low frequency electrical stimulation of the motor cortex (LFSMC).

## Methods

Experiments were approved by the Animal Care Committee of ULB. We made all efforts to reduce animal suffering as much as possible and to use the minimal number of animals. Adult Wistar rats (*n* = 11; weight between 240 and 390 g) were anaesthetized with chloral hydrate (400 mg/kg i.p., followed by a continuous infusion to obtain a steady-state anesthesia after about 15 min and reproducible motor evoked potentials MEPs; CMA micropump, CMA, Sweden) before the beginning of the surgical procedure [8]. Rats were put in a stereotaxic apparatus (Kaps, Germany). Scalp was shaved and cut sagitally. The tissue overlying the cranium was removed (epicranial stimulation to obtain corticomotor responses and epidural stimulation for cDCS; see below). Body temperature was maintained between 36.0 and 37.5 °C. Indeed, this parameter is critical for the activity of glutamatergic pathways [9].

### Experimental protocol

The following protocol was applied:

A. Baseline Measurements (MEPs and conditioned corticomotor responses)
B. LFSMC (T0–T10 min)
C. Measurements post-LFSMC (T20 min)
D. Measurements post-LFSMC (T45 min)
E. cDCS + LFSMC (T50–T60 min)
F. Measurements post-cDCS/LFSMC (T70 min)

### Motor threshold (MT) and Motor evoked potentials (MEPs)

We first determined the “hot spot” of the left gastrocnemius muscle by stimulating the right motor cortex using a mapping procedure (matrix of 6 × 9 sites) [10]. Stimulation was applied every mm in the sagittal axis and every 0.5 mm in the coronal axis (epicranial stimulation). We used a successive point-by-point stimulation method with monophasic pulses. The duration of electrical stimuli (square waves) was 1 msec (NeuroMax 4, Xltek, Canada). The right motor cortex was stimulated at an intensity of 130 % of the motor threshold MT (defined as the minimal intensity eliciting at least 5 out of 10 evoked responses with an amplitude >20 μV). We measured peak-to-peak amplitudes of MEPs (sets of 10 corticomotor responses were considered to compute the mean responses). We inserted subcutaneous needle electrodes (Technomed 017K25) in left gastrocnemius muscle to record MEPs. Impedance was maintained below 5 KOhms.

### Conditioned corticomotor responses

The conditioning stimulus (DS70 stimulator, Digitimer, UK) was delivered in the left sciatic nerve (stimulation at a distance of about 16 mm laterally from midline; intensity of stimulation eliciting a small twitch of the hindlimb) at an inter-stimulus interval (ISI) of 6 msec before application of a test stimulus on right motor cortex. Such short ISIs are associated with an afferent facilitation whereas long ISIs are associated with an afferent inhibition [10].

### Low-frequency electrical stimulation to the motor cortex (LFSMC)

For LFSMC, we administered squared pulses (duration: 1 msec) at an intensity corresponding to the MT at a frequency of 1 Hz during 10 min (600 stimuli) over the right motor cortex after the following baseline measurements: (1) a set of 10 MEPs without conditioning, followed by (2) a set of 10 duos of MEPs for the conditioning paradigm [10]. LFSMC was applied from T0 min to T10 min (current supplied by a constant current stimulator A310-A365, World Precision Instruments, UK). rTMS at the frequency of 1 Hz and at an intensity corresponding to MT is known to elicit a strong reduction in motor cortex excitability [5]. Durations of 5 to 20 min have been applied in rats by other groups using rTMS [5]. We first observed (in 3 rats) that the depression of MEPs was maximal from T10 to T30 min. The depression lasted about 25 min (amplitudes of MEPs returned to baseline values at about T35 min). We repeated the measurements of MEPs (10 MEPs without conditioning and 10 duos of MEPs in the conditioning paradigm) 10 min after the end of application of LFSMC (T20 min). We selected to combine cDCS (see next section) with LFSMC 50 min (from T50 to T60 min) after the beginning of LFSMC, when the excitability of the motor cortex had returned to baseline values.

### Cathodal transcranial direct current stimulation (cDCS) over the cerebellum

cDCS was applied over left cerebellar hemisphere during 10 min in conjunction with the application of LFSMC applied over the right motor cortex (from T50 to T60 min). The method to apply cDCS has been reported earlier [11]. This is based on the study of Fregni et al. [12]. The anode (low impedance metallic electrode with a diameter of 0.8 mm) was fixed 5 mm anterior to the bregma in right supraorbital region, inserted epicranially. A small plastic jacket was fixed over left cerebellar hemisphere with dental cement and filled with saline solution (0.9 % NaCl) to obtain a contact area of 7.1 mm^2^. The cathode was applied epidurally over the left cerebellar hemisphere. cDCS was applied directly onto the dura to ensure a defined contact area over the cerebellar cortex. The after-effects of cDCS last about 55–65 min [11]. We previously showed that cDCS does not change the amplitudes of MEPs, redistributes corticomotor maps and does not modify the afferent inhibition [11]. Measurements of MEPs (10 MEPs without conditioning and 10 duos) were repeated at T70 min. At the end of the experiments, an overdose of chloral hydrate (1000 mg/kg i.p.) was administered. Following decapitation, brains were extracted and examined under a microscope to exclude local lesions or bleeding.

### Statistical analysis

Statistical analysis was performed using Sigma Stat (Jandel Scientific, Germany). The normality of data was assessed using the Kolmogorov-Smirnov test. We compared the amplitudes of MEPs without conditioning before LFSMC (baseline; T0 min), after LFSMC (T20 min), at T45 min (to confirm return to baseline values’ range) and after the combination cDCS/LFSMC (at T70 min) using the Friedman repeated measures analysis of variance on ranks, followed by pairwise multiple comparison procedures with the Tukey test. We compared the conditioned responses (afferent facilitation: ratio of conditioned response CR by unconditioned response UR) in the 4 recording times (at T0, T20, T45, T70 min) using the repeated measures analysis of variance, followed by the Tukey test. Despite the results of the normality assessment for conditioned responses, we also computed a Friedman repeated measures analysis of variance on ranks followed by the Tukey test given our sample size. Statistical significance was set at *p* = 0.05.

## Results

**Fig. 1:**
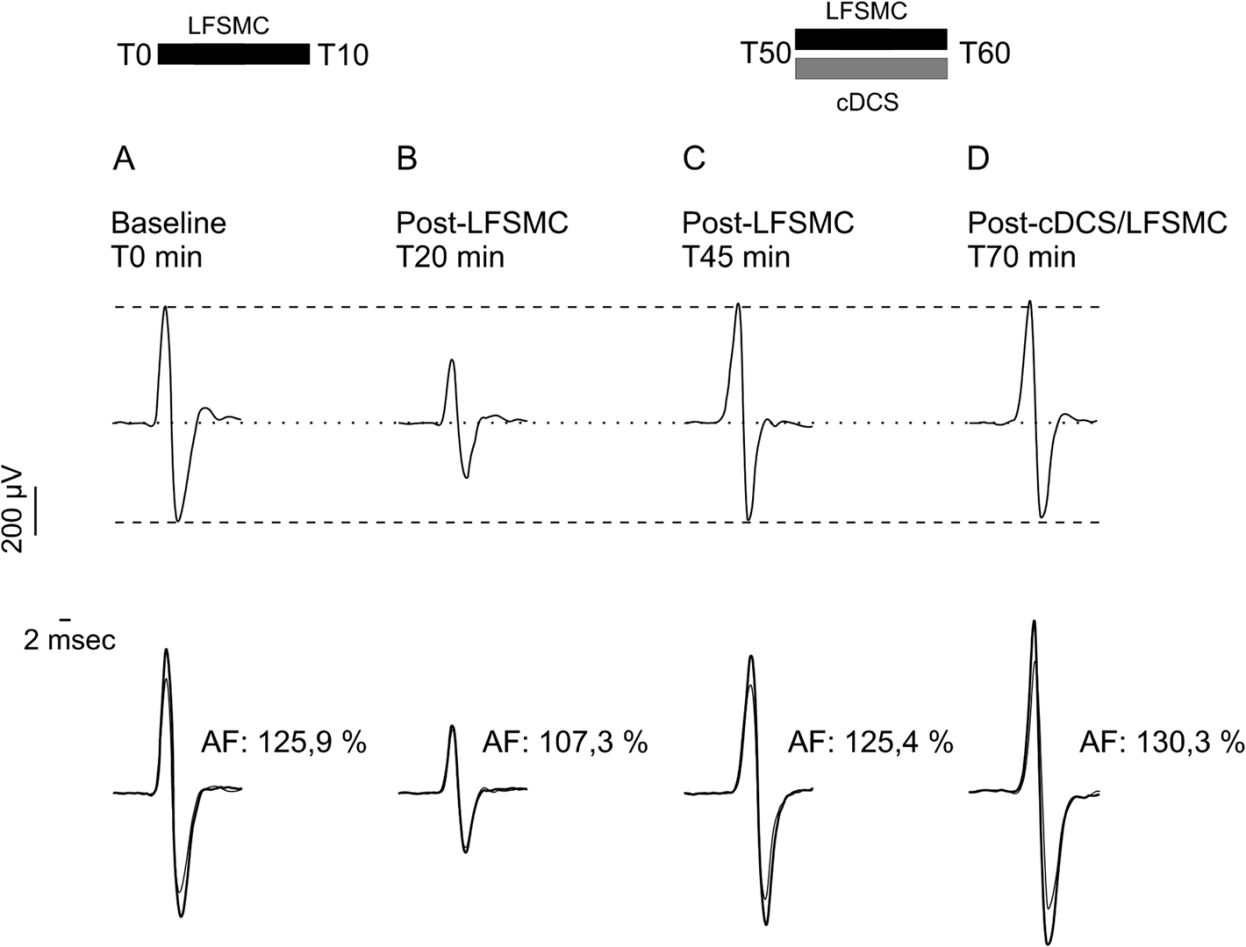
*Top panels*: example of averaged corticomotor response (MEP: motor evoked potential; averaging of 10 responses) evoked in left gastrocnemius muscle (stimulation of right motor cortex) at baseline (*A*, T0 min), 10 min after application of low frequency electrical stimulation of the motor cortex (*B*: post-LFSMC; duration of LFSMC: 10 min from T0 to T10 min), 45 min after baseline recording when the excitability of the motor cortex has returned to basal state (*C*: T45 min), after application of combined cDCS (cathodal DCS of the cerebellum) and LFSMC from T50 to T60 min (*D*: T70 min). *Bottom panels*: superimposition of averaged unconditioned MEPs (*thin traces*) and averaged MEPs with a conditioning stimulus (*thick traces*) during the paradigm of afferent facilitation (AF). Values of AF are given near the corresponding duos of traces

We did not observe lesions induced by electrical stimulation or bleeding in the motor cortex or in the cerebellum. The amplitudes of MEPs were significantly depressed by LFSMC. However, this LFSMC-induced depression was antagonized by cDCS of the cerebellum. This is illustrated in Fig. 1 (top panels). The afferent facilitation (assessed by the ratios of CR divided by UR) was depressed by LFSMC (Fig. 1, bottom panels). cDSC of the cerebellum antagonized the effects of LFSMC and even unbalanced the effects of LFSMC.

**Fig. 2:**
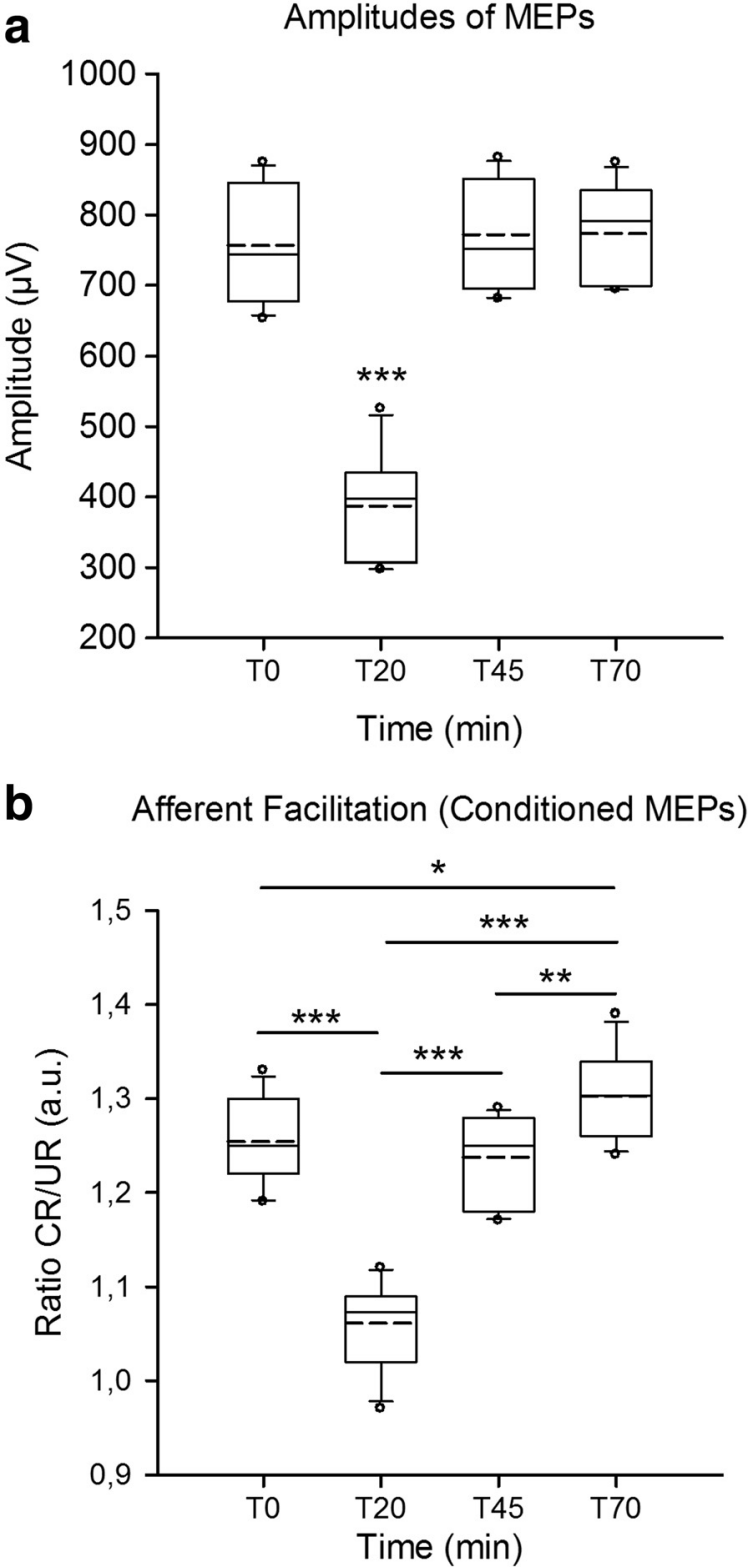
**a**: box and whisker plots of amplitudes of MEPs expressed in μV. ***: *p* < 0.001 as compared to T0, T45 and T70 min. **b**: box and whisker plots of afferent facilitation. Ratios of conditioned (CR)/unconditioned (UR) are shown at T0, T20, T45 and T70 min. Ratios are expressed in arbitrary units (a.u.). Medians (*continuous lines*), *dotted lines* (mean values) and outliers are illustrated. *: *p* < 0.05, **: *p* < 0.01, ***: *p* < 0.001

For the amplitudes of corticomotor responses, normality test failed (*p* < 0.05). The Friedman test showed a statistically significant difference between the 4 conditions (Fig. 2a; *p* < 0.001, coefficient of concordance of 0.763 and average rank r of 0.739). Tukey test showed that amplitudes of MEPs were significantly smaller at T20 min as compared to baseline (T0 min), T45 min and T70 min (*p* < 0.05). Normality test passed for the conditioned corticomotor responses in the paradigm of afferent facilitation (*p* = 0.119; equal variance test: *p* = 0.115). The analysis of variance showed a statistically significant difference between the 4 conditions (Fig. 2b; *F* = 78,92 with *p* < 0.001, coefficient of concordance of 0.878 and average rank r of 0.866). Post-hoc multiple comparisons revealed that ratios of CR divided by UR were significantly smaller at T20 min as compared to baseline, T45 min and T70 min (*p* < 0.001). cDCS entirely reverted the effects of LFSMC and even surpassed these effects. Indeed, ratios were significantly greater at T70 min as compared to T0 min (*p* = 0.034), and at T70 min as compared to T45 min (*p* = 0.003). There was no statistical difference between T0 min and T45 min (*p* = 0.751), confirming that the excitability of the motor cortex had returned to the range of baseline values.^1^

## Discussion

We provide the first experimental evidence that cDCS of the cerebellum antagonizes the strong inhibitory effect exerted by LFSMC applied over the contralateral motor cortex, expanding our understanding of the numerous and complex interactions between motor cortex and cerebellum [13]. We focused on the gastrocnemius muscle and cannot extrapolate the results to other limb muscles.

At this stage, we cannot distinguish between (a) an antagonistic effect of cDCS on the plasticity related changes induced by LFSMC, (b) an additive modulation of corticomotoneuronal output, and (c) a combination of the two mechanisms. Further studies are required, including single-cell recordings in the various layers of the cerebral cortex, especially recordings of inhibitory interneurons and pyramidal cells in the deep cortical layers. In addition, an effect upon extra-pyramidal pathways and/or spinal cord cannot be ruled out. Both the ratios Mean F/Mean M response and the persistence of F waves are significantly increased in the ipsilateral gastrocnemius muscle after application of cDCS of the cerebellum alone [11]. By contrast, cDCS does not modify the amplitudes of H reflex. We have demonstrated in a previous study that LFRSMC at an intensity of 130 % of MT (frequency of 1 Hz) changes the inter-hemispheric inhibition (IHI) but does not modify the excitability of the anterior horn motoneurons pool [14]. Therefore, repetitive stimulation of M1 at 1 Hz at an intensity of MT is unlikely to change the excitability of the spinal cord. In rats, pyramidal neurons and GABAergic interneurons of deep cortical layers receive directly the inter-hemispheric information [15]. Pyramidal neurons of layer VI respond monosynaptically to callosal stimulation [16]. This argues for a direct effect of low frequency stimulation upon the cerebral cortex itself. Layer VI pyramidal cells have wide projections towards other cortical areas [16]. Callosal information contributes to a bilateral corticothalamic integration by modulating the activity of inhibitory interneurons involved in cellular plasticity [15]. The interhemispheric inhibition (IHI) is a key-mechanism for the balance of activities between the 2 hemispheres [14]. Because the anode was fixed anterior to the bregma in right supraorbital region for the cDCS, a contribution of callosal pathways should be considered in our experiment. Moreover, the study of Fregni et al. on cortical spreading depression (a wave of neuronal depolarization propagating across the cortical surface) in rats argues for a direct effect of repetitive electrical stimulation at 1 Hz upon the cerebral cortex [12].

One limitation of the study is the use of continuous anaesthesia, a factor which might impact on the mechanisms of plasticity of the brain and which might influence our results. In our model, continuous infusion of chloral hydrate is required to obtain reproducible corticomotor responses. Although the half-life of chloral hydrate is short (a few minutes), the half-lives of the metabolites (trichloroethanol TCE and trichloroacetic acid TCA) are longer (up to 60 h) [17]. The mechanisms of action of chloral hydrate remain poorly understood but are known to involve GABAergic pathways, which are implicated in brain excitability and plasticity, especially for long-term plasticity [18]. Ideally, brain plasticity should be investigated without administration of anesthetic agents. This remains a major challenge for the experiments on corticomotor responses in vivo in rodents.

The excitability of the motor cortex can be tuned by acting directly on the motor cortex such as applying LFSMC, or by acting on anatomical structures targetting the motor cortex such as the prefrontal cortex, the sensory cortex or thalamic nuclei. For instance, the motor commands from M1 can be shaped by a modulation of the activity of rFr2 (prefrontal area, the equivalent of the premotor/supplementary motor areas in primates) [17–20]. The mechanism of afferent facilitation is enhanced if preceded by trains of electrical stimulation applied over rFr2 [10]. It has also been shown that repetitive somatosensory peripheral stimulation increases the excitability of the motor cortex and that an intact cerebellum is required for this form of short-term brain plasticity [21]. The activity of thalamic nuclei can be modified by acting on the cerebellar circuitry [22, 23]. The cerebello-dentato-thalamo-cortical pathway is a major actor in the anatomo-functional dialogue between the motor cortex and the cerebellum. The cerebellum is particularly responsive to electrical stimulation for anatomical and physiological reasons [24–26]. Modelling studies of cerebellar DCS indicate that the electric field (E) and the current density (J) spatial distributions occur mainly in the cerebellar cortex, with negligible spreads towards the brainstem [25]. Studies on cerebellar cortex ablation have confirmed the importance of Purkinje cell firings upon the discharges of cerebellar nuclei [27]. Such lesions cause a considerable increase in the background firing and cancel the pauses in discharges occurring in responses induced by somatosensory stimuli.

We have discussed previously the main anatomical pathways involved in the modulation of corticomotoneuronal output and the effects of rTMS on the excitability of the motor cortex [10]. Whereas transcranial electrical stimulation excites directly the axons of pyramidal neurons and generate direct (D) waves, TMS evokes several volleys of corticospinal activity : D-waves from direct axonal activation and later waves (I-waves) resulting from activation of mono- and polysynaptic inputs to pyramidal neurons [28, 29]. TMS excites the pyramidal neurons transsynaptically [29]. With both transcranial electrical stimulation and TMS, high-frequency synchronized descending volleys of activity are recorded in the epidural space [30–32]. However, there is still some debate on the synaptic mechanisms at the origin of I waves [33].

aDCS and cDCS of the cerebellum cannot just be considered as having pure opposite effects. Using an ISI of 45 msec, aDCS of the cerebellum enhances the afferent inhibition of conditioned corticomotor responses, unlike cDCS which has no significant impact on the afferent inhibition [11]. aDCS decreases the amplitude of corticomotor responses and changes the representation pattern of limb muscles over the motor cortex. A “focusing effect” is observed, with a concentration of the highest motor responses around the hot spot. In this case, an opposite effect occurs with cDCS.

MEP suppression by 1Hz rTMS has been demonstrated in rats under general anesthesia [5]. Several authors consider that repetitive electrical stimulation of the cerebral cortex in the rat mimics the effects of magnetic stimulation [12]. A long-term depression (LTD)-type plasticity is suggested to explain MEP suppression. Indeed, the effects of rTMS are known (1) to be frequency-dependent, (2) to outlast the period of stimulation, and (3) to rely on NMDA pathways [5, 34, 35]. Our results show that the neuromodulation of cerebellar activity by cDCS exerts powerful remote effects on the LTD-like plasticity induced by LFSMC. Therefore, the 2 techniques appear to compete in terms of consequences on motor cortex excitability. Regarding rTMS, its direct application over the cerebellum tunes the activity of contralateral motor cortex. One Hz rTMS over the cerebellar cortex increases intracortical facilitation (ICF) at the level of contralateral M1, and low-frequency cerebellar rTMS trains affect motor intracortical excitability beyond the application of the train [36]. ICF is depressed in hemicerebellectomized rats but remains responsive to trains of stimulations applied over the prefrontal cortex [10].

What are the implications in the field of clinical ataxiology? We propose the following potential therapeutical applications of DCS in cerebellar patients, still deserving further confirmations in specific clinical studies. Lesions or dysfunction of cerebellar nuclei induce a depression of contralateral motor cortex excitability which can be reverted either by aDCS of the motor cortex [37, 38] or by cDCS of the cerebellum which disinhibits cerebellar nuclei. Examples of dysfunction of cerebellar nuclei are compression by tumors of the surrounding white matter or hydrocephalus, intoxications [37, 39], spinocerebellar ataxia type 3 (SCA3) or Friedreich ataxia which are associated with a nuclear grumose degeneration [40, 41], calcium deposits [42, 43]. It is currently unclear whether the recently reported deposits of gadolinium in cerebellar nuclei of patients having received multiple administrations will be associated with very slowly evolving cerebellar deficits (motor, cognitive and/or affective) or will remain clinically silent for life [44]. Lesions of the cerebellar cortex (cerebellitis, cerebellar cortical atrophy) lead to a disinhibition of cerebellar nuclei and overactivity of contralateral motor cortex. Application of LFSMC or aDCS of the cerebellum could be administered to regulate motor output. It was shown recently that aDCS improves ataxias associated with cerebellar atrophy [45]. Disorders combining a pathology of the cerebellum and hyperexcitability of the motor cortex could benefit from combinations of LFSMC and DCS of the cerebellum. One example is familial cortical myoclonic tremor with epilepsy [46, 47]. Finally, the modulation of the motor cortex excitability is considered as an early change before structural plasticity [48, 49] and therefore combinations of LFSMC/DCS of the cerebellum may find future applications in the attempts to influence sensorimotor learning with a clinical perspective in mind. On a broader perspective, the remote supervision of LTD-like mechanisms in the cerebral cortex might be a mean to act on sensori-motor learning disorders and could be envisioned as a novel tool to appreciate the functional preservation of the cerebellar projections towards the primary motor cortex, complementing the previously reported properties of DCS in the detection of very early lesions in the cerebral cortex [50] and adding another electrophysiological tool to assess cerebellum-brain interactions [51]. The hypothesis that the cerebellum filters or processes time-specific incoming sensory volleys in order to influence the plasticity of the motor cortex is reinforced by findings of impaired long-term potentiation (LTP)-like effects during paired associative stimulation [52]. Overall, cerebellum appears as key-actor for the supervision of remote plasticity in the motor cortex.

## Conclusion

This is the first demonstration that cDCS of the cerebellum modulates the effects of LFSMC upon the excitability of motor circuits. Our results potentially open future applications for a cerebellar remote control of LFSMC-induced neuroplasticity in vivo.

## Footnotes

1 Given the sample size, a Friedman repeated measures analysis of variance on ranks was also applied despite the values of the normality assessment. The *p* value (the 4 conditions being considered) was <0.001. Tukey test showed that values at T70 min were greater than values at T45 min and T20 min, and that values at T0 min were greater than values at T20 min. However, values at T20 min are similar to values at T45 min. In addition, values at T70 min were similar to values at T0 min.
